# Workload distribution in wild Damaraland mole-rat groups

**DOI:** 10.1098/rstb.2023.0276

**Published:** 2025-03-20

**Authors:** Shay Rotics, Hanna M. Bensch, Yehezkel S. Resheff, Tim Clutton-Brock, Markus Zöttl

**Affiliations:** ^1^ School of Zoology, Faculty of Life Sciences, and the Steinhardt Museum of Natural History, Tel Aviv University, Tel Aviv 6997801, Israel; ^2^ Department of Zoology, University of Cambridge, Cambridge CB2 3EJ, UK; ^3^ Kalahari Research Centre, Kuruman River Reserve, Van Zylsrus 8467, South Africa; ^4^ Department of Biology and Environmental Science, Centre for Ecology and Evolution in Microbial Model Systems (EEMIS), Linnaeus University, Kalmar 391 82, Sweden; ^5^ The Hebrew University of Jerusalem, Business School, Jerusalem 91905, Israel; ^6^ Department of Zoology, Mammal Research Institute, University of Pretoria, RSA, Pretoria 0028, South Africa

**Keywords:** cooperative breeding, work division, Mammals, eusociality, vertebrate societies, caste system, body acceleration logging, mole-rats

## Abstract

The social organization of Damaraland and naked mole-rats is often suggested to resemble the societies of eusocial insects more closely than that of any other vertebrate. Eusocial insects feature queens that hardly contribute to the workforce, and specialized worker castes. However, in Damaraland and naked mole-rats, which live in family groups with a single breeding pair and multiple non-breeding helpers, the work division is still unclear. Previous studies, largely confined to laboratory settings, could not quantify their primary cooperative behaviour, which is digging extensive foraging tunnels. Here, we studied the distribution of workload in 11 wild Damaraland mole-rat groups, using body acceleration loggers to evaluate behavioural time budgets of 86 individuals. We found behavioural differences between breeders and non-breeders that emerged with increases in group size, such that in large groups, breeders spent less time digging, more time resting, and were overall less active than non-breeders. We did not find any indication of a caste system among non-breeders, though the amount of time individuals spent digging varied with age and sex. Overall, the lower contribution by breeders to the group’s workload is a pattern rarely observed in other cooperative vertebrates; nevertheless, the lack of evidence for castes suggests that eusociality may be limited to invertebrates.

This article is part of the theme issue ‘Division of labour as key driver of social evolution’.

## 1. Introduction

The complex societies of eusocial insects, such as termites, ants and honeybees, have fascinated biologists and raised the question of whether similar societies exist among vertebrates. It has often been suggested that the societies of two African mole-rat species, the Damaraland mole-rat (*Fukomys damarensis*) and the naked mole-rat (*Heterocephalus glaber*), are more closely comparable to those of eusocial insects than those of any other vertebrate [1–5]. Similar to termites, both of these species live underground in family groups with multiple non-breeding helpers and a single breeding pair [6–9], and maintain underground nests and communal food stores [6–9]. In addition, both species display other similarities with eusocial insects that are uncommon among vertebrates, including extended longevity of breeders compared with non-breeders [10,11], virtually complete reproductive suppression of subordinate females while in their natal groups [12–15] and the elongation of the bodies of breeding females [16–19]. Yet it is noteworthy that unlike in some eusocial insects, subordinate mole-rat females will start to reproduce upon separation from their natal group [14,20] and breeding mole-rat females' body elongation is not an innate morphological divergence but probably a consequence of pregnancy or dominance status [18,19].

Given the similarities in social structure, it is important to identify whether division of workload in mole-rat societies more closely resembles that of other singular cooperative breeding vertebrates or mirrors the complex social systems of eusocial insects. In eusocial insects, there is an almost complete division between the queen and workers into reproduction (the queen) and colony labour like foraging and brood care (the workers), sometimes with specialization of workers on different tasks (castes of workers) [21]. By contrast, in most cooperatively breeding vertebrates there is division in reproduction (breeders versus non-breeders) but little to no difference in cooperative labour or specializations [22]. Previous essays have emphasized behavioural differences among group members as a key to classifying these societies [22,23]. According to Crespi and Yanega [23], the key characteristic separating eusociality from cooperative breeding is the occurrence of castes, defined as ‘groups of individuals that become irreversibly behaviourally distinct prior to reproductive maturity’. Additionally, a recent review emphasized the importance of studying workload differences and task specializations for systematic understanding of cooperative vertebrate societies [22].

For social mole-rats, however, it is still unclear how the work is divided among group members, and to advance our understanding of vertebrate sociality, we need to pinpoint its place on a continuum of work division complexity between eusocial insects and cooperative breeding vertebrates. More specifically, we need to understand whether Damaraland and naked mole-rats display lower workload of breeders than non-breeders and a separation of non-breeders into specialized castes, as in eusocial insects [24,25], or whether they display only lower workload of breeders as in primitively social insects and a small fraction of cooperative breeding vertebrates (e.g. meerkats (*suricata suricatta*): [26], paper wasps (*polistes*): [27]; or else show neither of these two social features as in most cooperative breeding vertebrates [22,28–34].

The lack of clarity regarding work division in Damaraland and naked mole-rats stems from their strictly subterranean lifestyle, making it unfeasible to observe their behaviour in the wild. Consequently, almost all our knowledge of their cooperative behaviour comes from laboratory studies, wherein their central cooperative activity is not represented adequately. The most demanding cooperative activity of social mole-rats is construction and maintenance of extensive networks of foraging tunnels used in search for patchily distributed tubers [6,35–37], which cannot be displayed in confined laboratory settings. This activity is cooperative by nature because the tubers are usually larger than one individual can consume and are fed on by all group members [37,38]. In the laboratory, mole-rats transfer sand or sawdust from one place to another and this is commonly referred to as ‘digging’ [39,40], and laboratory studies have shown that breeders contribute less than non-breeders to the transfer of sand and to other cooperative activities [16,40,41]. However, this sand transfer behaviour in the laboratory requires less energy than digging in the hard soil in the wild and the unlimited food supply in the laboratory makes it less necessary. Hence, the validity of generalizing from these lab findings to the wild is questionable.

There is also an uncertainty regarding the existence of a caste system in these social mole-rats owing to inconsistent findings [5,42]. Early studies have suggested that both in Damaraland and naked mole-rat groups, non-breeders can be allocated to specialized, discrete castes that differ in body size and work frequency [1,4,41,43,44], where larger-bodied individuals work less frequently and possibly form a dispersers caste [4,45]. However, more recent laboratory studies have not found indications of discrete castes in either of these two mole-rats species and, instead, suggest that individual work frequency varies continuously with age [39,46–48]. This is an important controversy, concerning whether the evolution of castes is possible in vertebrates, which can be informed by examining the work division in wild mole-rat groups.

In this study, we investigated the behaviour of Damaraland mole-rats in a natural population in the southern Kalahari. Damaraland mole-rats live in groups of variable sizes, with an average of nine individuals [20,49,50]. Groups are typically nuclear families, consisting of one unrelated breeding pair and non-breeding offspring of both sexes that stay in the group for around 2−3 years before dispersal [2,7,20,49,51]. Dispersal is risky and most individuals do not survive this phase to attain a breeding role [20,51]. A female’s route to reproduction mostly involves founding a new group, after dispersing solitarily into a new territory and then being found by a dispersing male [20]; however, occasionally females can inherit a breeding role after the disappearance of her group's previous breeding female [20,51]. For males, reproduction is attained mostly by founding a new group, and less frequently by immigrating into an established one [20,51].

To study the Damaraland mole-rats' underground behaviour, and particularly the work division between group members, we used tiny body-acceleration loggers. The body-acceleration records provided estimates of the individual behavioural time budgets, particularly digging activities and resting time (using supervised machine learning classification [52], as well as estimates of general activity level (based on Overall Dynamic Body Acceleration [53])). Using these estimates, we first investigated whether breeders contribute less to tunnel excavating activities (cooperative foraging) and rest more than non-breeders and whether these contrasts increase with group size, as they do in captive colonies [40]. There are cooperative behaviours like nest building or carrying pups back to the nest [8,39] that were not addressed here owing to limitations in inferring particularly rare behaviours from body acceleration data. We also examine feeding posture differences that imply whether breeders use the communal food store more frequently than non-breeders (see §2 for explanation). We further explored whether there is a division of labour among non-breeders that resembles the caste systems of eusocial insects. To do this, we examined three predictions that are expected to be met if castes are present among non-breeding mole-rats following Zöttl *et al*. [39]. First, we tested for a bi- or multimodal distribution in the digging efforts among non-breeders, reflecting a division between individuals of different castes. Second, we checked whether individuals specialize in specific tasks, leading to negative correlations between individual contributions to different cooperative activities. Third, we tested whether individuals' cooperative contributions are negatively correlated to their growth rate. This last prediction is based on earlier mole-rat studies which implicitly suggested that smaller, slow-growing individuals belong to a ‘frequent worker’ caste that invests more in cooperative work and specializes on lifelong philopatry [4,45,54]. Finally, we explored the effects of sex and age on digging behaviour and activity level.

## 2. Methods

### (a) Study site and data collection

The study was conducted between September 2018 and November 2019 at the Kuruman River reserve, South Africa, on a mole-rat population that has been monitored in this site since October 2013 [20,50]. All individuals in this population were recaptured approximately every six months using modified Hickman traps, baited with sweet potato and positioned into their tunnel systems. At first capture, individuals were implanted with a subcutaneous passive integrated transponder microchip (Trovan, DorsetID, The Netherlands) and sexed by their genitals. Subsequently, on every capture, body mass (g) was recorded. Breeding females were identified from their perforated vagina and prominent teats [43,55]. Breeding males were identified through longitudinal capture records because these are the only large males found in breeding groups from group start or first group capture, and for a period of more than 2 years [51,56]. Age was estimated based on animal body mass at first capture and body mass growth curve of the population computed using *interval equations* [57,58] (see electronic supplementary material for explanation).

From September 2018 to October 2019, we fitted collars with body-acceleration loggers (axy4 loggers, Technosmart Ltd, Rome, Italy) to 96 Damaraland mole-rats from 11 groups. The collar was made of 0.81 mm nylon-coated wire (TECNI Ltd, UK) that was threaded into a 1.6 mm, 2 : 1 heat-shrink tube to reduce discomfort for the animal and was closed using an aluminium or copper ferrule (TECNI Ltd, UK; electronic supplementary material, figure S1). Collars with acceleration loggers were fitted to the animals under isoflurane anaesthesia and weighed 3 g in total. To keep this weight below 5% of animal weight, in each group only animals above 60 g were collared (average weight of collared individuals was 106 ± 31 g). Mole-rats below 60 g constituted 16% of individuals in our trapped groups, and are juveniles/sub-adults of less than *ca* four months of age [59], which according to laboratory observations contribute very little to cooperative behaviours [39]. Groups were collared only if the breeding female was not identified as pregnant or lactating based on visual inspection under anaesthesia. After individuals were collared, the groups were released back to the wild for three weeks, after which they were re-trapped for collar removal and released back to the wild again. We successfully re-trapped 92 individuals for collar retrieval, from which valid data were downloaded from 86 loggers; the other loggers did not provide data mostly owing to damage from mole-rat bites. All the field work was conducted with approval from the University of Pretoria Animal Ethics Committee and The Northern Cape Nature Conservation (permits: EC089−12, SOP−004−13, EC059−18).

### (b) Estimating behaviour from body acceleration (ACC) data

The loggers recorded acceleration measurements (with 10 bit data resolution) continuously at 25 Hz in each of the three perpendicular axes. To examine an individual’s activity, overall dynamic body-acceleration (ODBA) was calculated. ODBA reflects the extent of body movements and is considered a valid proxy of activity-related energy expenditure [53,60] and activity level [61,62]. ODBA was calculated per 2 s acceleration bout (consists of 50 acceleration measurements per each of the three axes) by subtracting each data point from a running average of 10 points and summing the resulting absolute values across axes [52].

Supervised machine learning (Gradient Boosting Classifier in Python Scikit-learn software [63]) was used to classify body-acceleration (ACC) records to behaviours. To train the classifier, 8023 ground-truth ACC records of known behaviours were obtained by videotaping collared mole-rats in captivity. The mole-rats were housed in a laboratory facility at the study site that includes systems of tunnels that mimic their underground habitat, and are built of mostly transparent tubes, enabling behavioural observations (see [39,40] for details). We recorded 57 ten-minutes videos from 16 collared individuals and labelled the behaviours (when clearly visible) using the ‘BORIS’ software [64]. In 19 of the video-recording sessions the mole-rat was placed within a dedicated, transparent, thin and vertical ‘digging box’ filled with moist sand that facilitated observing the digging activities (electronic supplementary material, figure S2). The machine learning training was conducted on 2 s segments of ACC records of a single behaviour. Shorter behaviours were omitted (*ca*. 6% of the labelled behaviours). For each of these segments, 50 data features were computed and used to train the classifier (electronic supplementary material, table S1). Fourteen different behaviours were regularly observed in the lab and were included in the classification, whereas rare behaviours were excluded (electronic supplementary material, table S2). Following the classification, the behaviours were grouped into four general categories: rest, excavating activities (digging, sweeping soil and back-kicking for soil tightening), movement (running, walking and food carrying) and other (electronic supplementary material, table S2), with an overall classification accuracy for these categories of 88% (electronic supplementary material, table S2). Our analyses focused on excavating activities, primarily consisting of digging and sweeping, which were correlated (Pearson’s *r* = 0.69, *t*_84_ = 8.7, *p* < 0.01), and resting. To a lesser extent we also examined eating, running and food carrying behaviours (accuracy: 0.77, 0.82, 0.76, respectively; for more details see electronic supplementary material, table S2). It is important to note that we used observations in captivity to train the machine learning model that was then used to classify behaviours in the wild, and this inevitably introduces some degree of uncertainty to our classified behaviours, which we tried to examine by testing the robustness of the results under different data scenarios (see details in electronic supplementary material).

Damaraland mole-rats feed mainly on a long tuber (gemsbok cucumber, *Acanthosicyos naudinianus*) that grows in the Kalahari [37], but occasionally also on smaller bulbs such as *Dipcadi gracilimum*. They can eat directly from tubers positioned vertically in the ground or transport food items (or pieces of them) to the group’s food store to be consumed later [6]. Eating directly from a tuber generates a more vertical body posture as the mole-rat leans against the tuber (electronic supplementary material, figure S3). In the laboratory, a cut-off in the mean *z*-axis acceleration value, reflecting the animal posture, discriminated clearly between mole-rats eating from a complete, vertically positioned tuber versus eating other smaller food items, which is characterized by a more horizontal body posture (cut-off: 4.9 ms^–2^ [half standard gravity]; accuracy: 99%; electronic supplementary material, figure S4). Following this distinction, we computed the individual proportion vertical eating from total eating (vertical eating records: mean *z*-axis < 4.9 ms^–2^; electronic supplementary material, figure S4) as an indication of eating habits in the wild, which relate to eating directly from tubers versus eating food items that might be more likely to originate from the group’s food storage.

### (c) Growth index

To test whether slow-growing non-breeders, which are presumably smaller for their age group, contribute more to excavating activities than fast-growing ones, we first computed the growth index per individual. This index was computed based on the individual body weights in its first two captures, as long as the first weight was < 90 g (which is roughly below one year of age) and the second weight was obtained between 3 and 8 months from the first mass (such weight records were available for 50 non-breeders out of 65). Individual growth was modelled as the difference between consecutive weights, using a generalized additive model (GAM) with smooth factor fitted for the first weight (by sex) to capture the nonlinear relationship between first weight and weight differences resulting from growth differences at different ages, and with the predictors: sex and days difference between consecutive weights. The residuals of this model served as the growth index of the individual, indicating whether it is below or above the predicted, average body mass growth curve of the population.

### (d) Data analysis

We obtained on average 17 days of continuous, 25 Hz body-acceleration data per individual (s.d. = ± 3.9). We focused on three behavioural metrics derived from this data: ODBA, proportion of time in excavating activities (digging and sweeping) and proportion of time resting. To investigate behavioural differences between breeders and non-breeders, we compared individual averages in each metric (separately) using generalized linear mixed models (GLMMs) with the predictors: breeding status, group size and sex, and we included the interaction between breeding status and group size, following Houslay *et al*. [40] who found such interaction in captivity. We also compared the eating posture, i.e. the proportion of vertical eating from total eating, between breeders and non-breeders. Group ID was included as a random factor in all analyses. Breeders are normally the oldest individuals in their group, therefore age was highly correlated with breeding status (*r* = 0.75) and was not included in the breeders versus non-breeders analyses.

Within non-breeders, we examined whether individual contributions to excavating activities followed a unimodal distribution. For this, we extracted the proportion of time spent in excavating activities per individual, computed their residuals after accounting for group ID and tested their distribution using Hartigans' Dip Test for Unimodality. We further checked for individual repeatability (intra-class correlation) in the daily time spent in excavating activities, examined using the ‘rptR’ package [65].

To test for task specialization among non-breeders, we tested the prediction that investment in different tasks should be negatively correlated within individuals; for this we examined the following behaviours: excavating activities, food carrying and walk-around. The latter was defined as running/walking bouts (of more than 5 s) that were not preceded by excavating bouts in the previous minute, as excavating work sequences normally involve running (the mole-rats dig, sweep the soil backwards and run back to digging point). Thus, ’walk-around’ was defined as locomotion unrelated to excavation, and may reflect tunnel patrolling behaviour. The relationship between each dyad of behaviours was examined using a GLMM while accounting for the random factor group ID (using beta-binominal error distribution); we report the associations by providing the marginal *R*^2^ [66] and the predictor’s *P* value.

We also tested the prediction that smaller, slowly growing non-breeders work more by examining the relationship between growth index and proportion time spent in excavating activities; this was tested using GLMM with the predictors: growth index, sex, group size, age (as a quadratic term) and the random factor group ID. Similarly, we modelled non-breeders’ growth index effects on their ODBA and proportion resting time.

### (e) Additional statistical notes

ODBA was analysed using GLMMs with gamma error distribution and proportions (relative time in excavating activities, relative time resting, vertical eating from total eating) were analyzed separately using GLMMs with beta binomial error distribution. Interactions and quadratic terms were fitted based on *a priori* expectations [40] or following preliminary diagnostic plotting that indicated their existence. To avoid multicollinearity, correlated predictors (*r* > 0.5) were not included in the same model (leaving 'age' out of models that included 'breeding status'), and variance inflation factor (VIF) values were always lower than 2. For all models, we verified that there was no heteroscedasticity and that the residuals fitted the expected error distribution using the DHARMa package in R [67]. Data were processed in Matlab (The MathWorks Inc., v. 9.0.0.341360 [2016]) and statistically analysed in R3.6 using glmmTMB [68] and GAM [69].

## 3. Results

### (a) Differences between breeders and non-breeders

**Figure 1.**
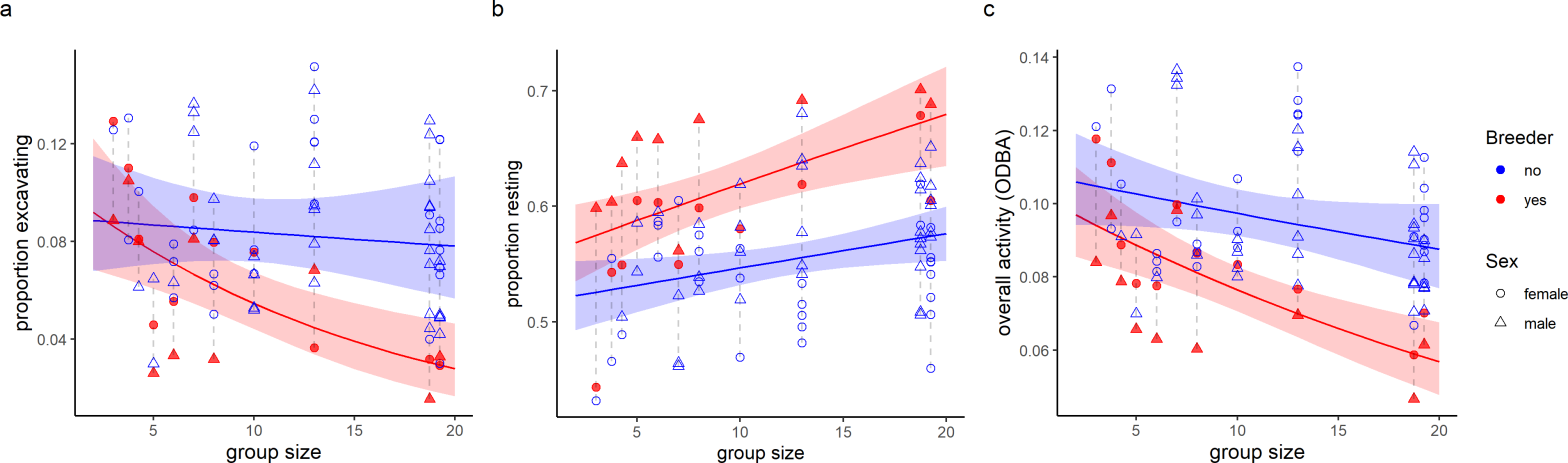
Differences between breeders and non-breeders in: (*a*) proportion of time in digging and sweeping activities (excavating), (*b*) proportion of time resting, (*c*) overall dynamic body acceleration (ODBA). Excavating and resting behaviours are predicted from body acceleration data using a machine learning model, whereas ODBA is a direct measure calculated from the acceleration data. Individual averages are displayed and dashed lines connect individuals of the same group (within which the comparison is most relevant). Lines and shaded areas mark the values ± CI_95_ as predicted by the models that are fully reported in table 1. After accounting for sex and the random factor group ID, breeding status effect and its interaction with group size are significant in all plots (GLMMs, *p* < 0.01, table 1). Colour corresponds to breeder status (blue = non-breeder, red = breeder); point shape corresponds to sex of the individual (circle = female, triangle = male).

**Figure 2.**
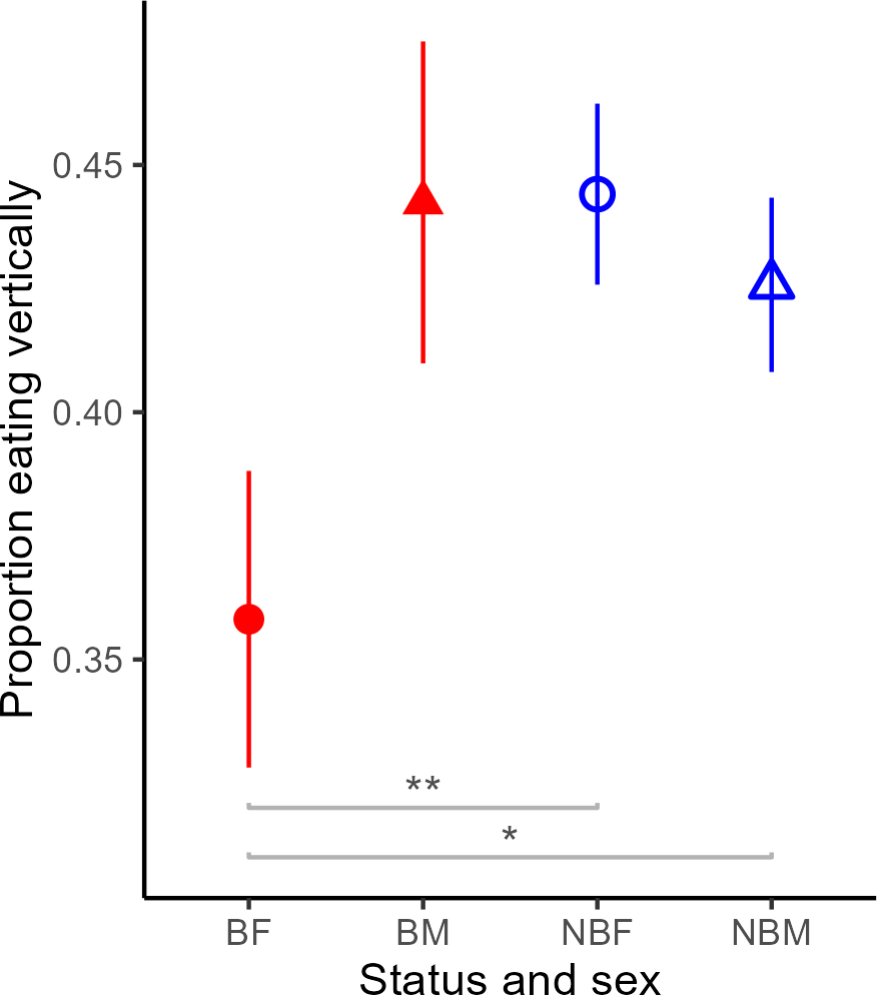
Differences in the proportions eating vertically (from total eating) between: breeding females (BF; *n* = 11), breeding males (BM; *n* = 10), non-breeding females (NBF; *n* = 29) and nonbreeding males (NBM; *n* = 36). Means ± SE are displayed. Significant differences are marked with asterisks (* = 0.04, ** = 0.01), based on a Tukey’s *post hoc* analysis on a GLMM model that is reported in electronic supplementary material, table S3. Breeding females’ eating postures differ from non-breeders in a way that can imply that they eat less from vertical tubers in the ground and more from detached tuber pieces, possibly from the group’s food storage.

Breeders in large groups of above nine individuals spent less time excavating and more time resting than non-breeders and were less active overall (lower ODBA), although similar contrasts did not occur in small groups where the contributions of breeders to digging increased (interaction: breeding status × group size, figure 1a–c, table 1). Breeding females, but not breeding males, also spent a lower proportion of their eating time in a vertical position than non-breeders of both sexes, suggesting that they may feed more often from the group’s food store than non-breeders (figure 2, electronic supplementary material, table S3).

**Table 1.** Behavioural differences between breeders and non-breeders.The table complements figure 1, presenting the results of three GLMMs conducted on the response variables: ODBA (gamma error distribution), proportion excavating activities (dig & sweep) and proportion resting (beta binomial error distributions), to examine behavioural differences between breeders and non-breeders. Each model included 86 individuals from 11 groups, group ID was included as a random factor and group size was scaled to the mean.

	ODBA				proportion time excavating				proportion time resting			
**parameter**	***β***	***SE***	***Z***	***P***	***β***	***SE***	***Z***	***P***	***β***	**SE**	***Z***	***P***
breeding status (breeder)	−0.274	0.035	−7.780	<0.001	−0.542	0.106	−5.112	<0.001	0.324	0.049	6.648	<0.001
group size	−0.007	0.007	−0.930	0.352	−0.005	0.015	−0.346	0.729	0.011	0.004	2.814	0.005
breeding status×group size	−0.020	0.006	−3.390	0.001	−0.066	0.018	−3.615	<0.001	0.018	0.008	2.196	0.028
sex (male)	−0.100	0.030	−3.370	0.001	−0.139	0.070	−1.976	0.048	0.181	0.039	4.670	<0.001

### (b) Testing castes-related predictions among non-breeders

**Figure 3.**
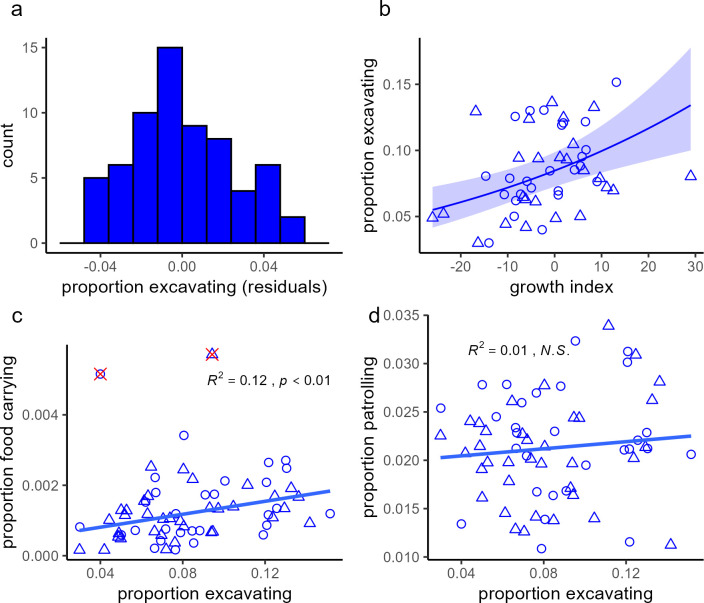
Examining castes-related predictions in non-breeders' workload. (*a*) Distribution of individual daily proportions of excavating activities (residuals after accounting for group ID), showing a unimodal shape (Hartigans' dip test for unimodality, *D* = 0.007, *p* = 0.99). (*b*) The effect of individual growth index, calculated as a residual from population body mass growth curve (higher values indicate faster growth; see §2), on proportion of excavating activities (dig and sweep); line and shaded area display the modelled effect ± CI_95_ (*p* < 0.001, see electronic supplementary material, table S5 for statistical details); circles and triangles mark data points of females and males, respectively. (*c*) Association between proportion of excavating activities and proportion of food carrying in non-breeders. *R*^2^ (marginal) and *P* value are provided (from examining the behaviours' relationship while accounting for group ID using a GLMM). Red × marks exclude outliers (verified with Grubbs’s test; association is marginally significant when outliers are retained; *R*^2^_marginal_ = 0.06, *p* = 0.05). (*d*) No association between proportion of excavating activities and proportion patrolling, defined as locomotion unrelated to excavating activity (see §2), which may reflect patrolling behaviour.

The relative time non-breeders spent in excavating activities followed a unimodal, normal distribution (Lilliefors normality test; *D* = 0.07, *p* = 0.53) rather than a multimodal distribution that would be expected if castes were present (figure 3a). Consistently, the activity level (ODBA) of non-breeders was also normally distributed (electronic supplementary material, figure S4). The relative time spent in excavating activities showed a significant individual repeatability, tested across the days of the study (*r*_rpt_ = 0.37, *p* < 0.001, likelihood-ratio test).

Slower-growing non-breeders spent less time excavating than faster-growing ones (figure 3b, electronic supplementary material, table S4). Slow-growing non-breeders also displayed lower overall activity (ODBA; GLMM; *β* = 0.008, s.e. = 0.002, *p* < 0.001, electronic supplementary material, table S4) and spent more time resting (GLMM; *β* = −0.006, SE = 0.003, *p* = 0.02, electronic supplementary material, table S4).

Across non-breeding individuals, there was no evidence of negative correlations between their contributions to different cooperative activities, indicating that they did not specialize in specific tasks. Contributions to different cooperative activities were either positively associated (excavating and food carrying, figure 3c) or not associated (excavating and walk-around: figure 3d; food carrying and walk-around: *R*^2^_marginal_ = 0.04, *p* = 0.60, GLMM).

### (c) Sex- and age-related differences

Females spent less time resting, were generally more active and showed a marginally significant tendency of spending more time excavating than males (table 1; but see a comment on the statistical robustness of this result in electronic supplementary material, table S4).

**Figure 4.**
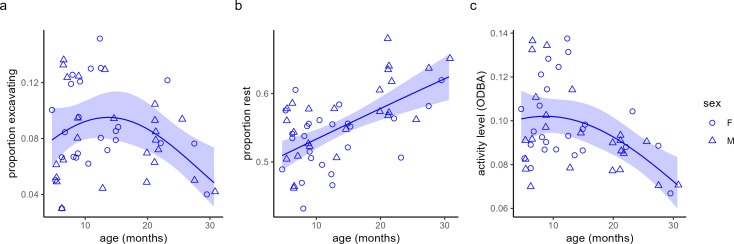
Age related variation in non-breeders' behaviour: (*a*) proportion time spent in excavating activities, (*b*) portion resting time, (*c*) overall activity (ODBA). The age was estimated based on the animal weight at first capture (see electronic supplementary material for details). Individual averages are displayed with shapes marking the individual sex (circle = female, triangle = male). Lines and shaded areas mark the values ± CI_95_ as predicted by the models that are fully reported in electronic supplementary material, table S4. After accounting for sex, growth index and the random factor group ID, the age effects in all plots were significant (GLMMs, *p* ≤ 0.01, electronic supplementary material, table S4).

Accounting for the effect of sex, non-breeders' age had a hump-shaped relationship with proportion of time spent excavating, peaking at around 14 months and declining subsequently (figure 4a). Additionally, resting time increased and activity level decreased with non-breeders' age (figure 4b,c).

## 4. Discussion

In this study, we used body-acceleration loggers to investigate the work division in wild Damaraland mole-rat groups to assess the extent to which their social organization resembles that of eusocial insects. Our results show that breeders contributed less to excavating activities, rested more and were less active (lower ODBA) than non-breeding group members in large groups, but these contrasts were absent in small groups (an interaction effect). We found no indication that non-breeders were divided into discrete castes that differ in body size and in contribution to excavating activities. These findings depict the Damaraland mole-rat’s social organization as featuring workload differences that benefit the breeders, which is rarely seen in cooperative vertebrates; however, unlike in eusocial insects, a caste system is not present.

Our results show that workload contrasts between breeders and non-breeders were a characteristic of large groups and were absent in small groups, presumably because breeding females increase their contributions to cooperative activities when fewer non-breeders are present to assist. Similar patterns have also been found in captive Damaraland mole-rats and in wild meerkats, where breeding females contribute less to cooperative activities than non-breeders and these contrasts increase with group size [40,70]. Correspondingly, the degree of division of labour in social insects has been positively linked to group size [71,72].

Our findings also showed that the breeding females' eating posture differs from that of non-breeders: the breeding females eat proportionally more in horizontal body posture than non-breeders. In a laboratory setup that we tested, eating in vertical body orientation reflects eating directly from a vertical tuber, and eating in a horizontal body posture reflects eating smaller tuber pieces that are likely to originate from the group’s food store (electronic supplementary material, figure S3). Therefore, our results imply that the breeding females are feeding proportionally less from *in situ* tubers, situated vertically in the ground, and proportionally more from the group’s food stores. Although some uncertainty regarding the interpretation of this finding remains, it may suggest that breeding females benefit relatively more than non-breeders from the group’s foraging efforts, which is in accordance with their reduced excavating activity. Comparable differences in feeding behaviour between breeding females and subordinates exist in African wild dogs (*lycaon pictus*), where breeding females remain at the breeding burrow after parturition and are provided with food brought from kills by other group members [73].

While reduced contributions to cooperative activities and the care of dependent young by breeding females (queens) are the norm in social insects, they are uncommon in vertebrates that breed cooperatively, where the breeding female typically contributes as much or more than the average non-breeding helper. From the 34 cooperative breeding mammals listed in Lukas and Clutton-Brock’s review [74], we found only three species other than Damaraland mole-rats in which breeders clearly contribute less than the average helper to the cooperative work. These were: meerkats (*suricata suricatta*) [70,75], dwarf mongooses (*helogale parvula*) [76] and naked mole-rats [41], while in most species from this list the *per capita* contributions of breeders to cooperative activities were as large or larger than those of non-breeders (e.g. golden and silver back jackal (*canis aureus, canis mesomelas*): [28], Common marmoset (*callithrix jacchus*): [29], Arctic fox (*vulpes lagopous*): [77], striped mouse (*rhabdomys pumilio*): [31], lion tamarins (*leontopithecus chrysomelas*): [78],cotton top tamarins (*saguinus oedipus*): [79], and in others, there were insufficient data to determine (see electronic supplementary material for details). Similarly, in cooperative breeding birds, breeders contribute as much or more to cooperative activities as non-breeders (electronic supplementary details in: [22,33]). There are a few equivocal cases in which some tasks are performed proportionally more by breeders and some by helpers, yet it is not clear if the breeders carry out the easier tasks, i.e. contribute less to the cooperative effort (these cases include: babysitting and hunting in African wild dogs (where the babysitter is fed by the hunters but gets to feed less [73], provisioning and infant carrying in tamarins [80,81] and shelter defence and shelter maintenance in cichlids [82]). In sum, the 40% lower digging effort by breeders compared with non-breeders that we find in large Damaraland mole-rat groups can be considered an outstanding degree of social workload division among vertebrates.

While the contributions of non-breeders to cooperative activities varied with their age and sex, we did not find any evidence for a caste system among non-breeders. They were not segregated into distinct groups based on their digging efforts, as indicated by the absence of a bi-modal workload distribution. Additionally, slow-growing, small-bodied non-breeders did not participate in work more frequently—contrary to previous suggestions that lighter individuals constitute a frequent worker caste [4]. Lastly, there was no evidence for task specialization, as individuals that contributed more to a specific task did not contribute less to another task. These patterns are consistent with recent laboratory studies in Damaraland mole-rats [39,46] and naked mole-rats [47]. As a number of other studies of cooperative breeding mammals and birds have shown, the contributions of non-breeders vary with their sex and age and contributions to different cooperative activities are usually positively correlated with each other [26,46,83,84], indicating that task specialization is probably rare in vertebrates [22]. The apparent lack of a caste system in Damaraland mole-rats indicates that they do not fall within Crespi and Yanega’s [23] definition of eusociality, and suggests that the similarities in social organization of Damaraland mole-rats and eusocial insects may have been overemphasized.

The social organization of Damaraland mole-rats closely resembles that of meerkats [85]. The two species are similar in group size and composition, dispersal age and reproductive skews [6,51,85] and they show very similar status, age and sex-related differences in cooperative behaviour [70,85]. The occurrence of relatively rare mammal society above (meerkats) and below (mole-rats) ground in the southern Kalahari supports the suggestion that the evolution of cooperative breeding systems is often associated with the occupation of arid, unpredictable environments [86,87].

Overall, our findings from the wild match previous findings from laboratory studies of Damaraland mole-rats, strengthening confidence in both sets of findings. These consistent findings include similar differences in work frequency between breeding females and non-breeders [40], positive correlation between growth and work frequency [46] and no indications of a caste system [39]. Even the average proportion of time spent digging (8%) corresponds closely to values observed in laboratory studies [39,46]. There were, however, also some dissimilar patterns such as differences in digging frequency between females and males—and between breeding males and non-breeders—that were found in the wild but not in the laboratory [40,46] and this warrants further investigation. Nonetheless, the overall consistency between findings from the wild, based on estimated behaviours from body acceleration records (using a machine-learning model trained with laboratory data) rather than on direct observations, and findings from laboratory studies where mole-rats were held in artificial tunnel systems with bi-hourly provision of new sand (e.g.: [8,40,46]), enhances confidence in both types of findings.

Many of the social species among mammals are rodents with fossorial lifestyle and revealing their underground behaviour would significantly expand our understanding of animal sociality. We believe that this is the first study to quantify workload distribution underground in strictly subterranean animals. Body-acceleration biologging is an excellent methodology for this as acceleration signals can be translated to basic behaviours, and recording them consumes low battery power, permitting light-weight loggers suitable for small subterranean species. We hope that the methodology implemented here will lead the way for more studies and discoveries on the elusive behaviour of animals underground.

## Data Availability

Data and main code are available as supplementary material. Supplementary material is available online [88].
